# Detection of *Echinococcus multilocularis* in repurposed environmental DNA samples from river water

**DOI:** 10.7717/peerj.15431

**Published:** 2023-06-14

**Authors:** Kensuke Mori, Akio Imamura, Itsuki Hirayama, Toshifumi Minamoto

**Affiliations:** 1 Graduate School of Human Development and Environment, Kobe University, Kobe, Hyogo, Japan; 2 Hokkaido University of Education, Sapporo, Hokkaido, Japan

**Keywords:** Environmental DNA, Echinococcus multilocularis, Repurposed eDNA samples, Zoonotic disease

## Abstract

Environmental DNA (eDNA) is an increasingly popular tool in biological and ecological studies. As a biproduct of its increasing use, large number of eDNA samples are being collected and stored, that potentially contain information of many non-target species. One potential use for these eDNA samples is a surveillance and early detection of pathogens and parasites that are otherwise difficult to detect. *Echinococcus multilocularis* is such a parasite with serious zoonotic concern, and whose range has been expanding. If eDNA samples from various studies can be repurposed in detecting the parasite, it can significantly reduce the costs and efforts in surveillance and early detection of the parasite. We designed and tested a new set of primer-probe for detecting *E. multilocularis* mitochondrial DNA in environmental medium. Using this primer-probe set, we conducted real-time PCR on repurposed eDNA samples collected from three streams flowing through an area of Japan endemic to the parasite. We detected the DNA of *E. multilocularis* in one of the 128 samples (0.78%). The discovery suggests that while detecting *E. multilocularis* using eDNA samples is possible, the rate of detection appear to be very low. However, given the naturally low prevalence of the parasite among wild hosts in endemic areas, the repurposed eDNAs may still be a valid option for surveillance in newly introduced areas with the reduced cost and efforts. Further work is needed to assess and improve the effectiveness of using eDNA for detection of *E. multilocularis*.

## Introduction

Environmental DNA (eDNA) is an increasingly popular tool for ecological studies, allowing researchers to assess presence of rare species in non-invasive manner at modest efforts and cost. While most eDNA samples are used for studying particular species or taxon of interest, the samples also contain genetic information of other taxa that co-inhabit the area (Thomsen et al., 2012; Valentini et al., 2016). These eDNA samples are potential troves of information for other researchers studying different taxonomic groups (Taberlet et al., 2012; Dysthe et al., 2018).

Environmental DNA can be particularly useful for detection of emerging or newly introduced pathogens and parasites that are difficult to locate due to their life style and small size (Bass et al., 2015). One example of an invasive parasite of serious concern is *Echinococcus multilocularis*, a causative agent of highly fatal zoonoses alveolar echinococcosis. Because of its high fatality, *E. multilocularis* was considered the most relevant food-borne parasite in Europe (Bouwknegt et al., 2018) and third most relevant worldwide (World Health Organization & Food and Agriculture Organization of the United Nations, 2014). While the parasite is naturally present across wide areas of northern hemisphere, it has expanded its range to new areas in recent decades (Gesy et al., 2013; Massolo et al., 2018; Santa et al., 2021). In Japan *E. multilocularis* was mostly restricted to northern island of Hokkaido, but recently it was discovered infecting several feral dogs captured in the central part of the main island of Honshu that is not adjacent to the endemic Hokkaido island, suggesting occurrences of local infections (Morishima et al., 2016). Because wildlife acts as natural reservoirs for the parasite, it is practically impossible to eradicate the parasite once it is established (Ito, Romig & Takahashi, 2003). Early detection and response are necessary if they are to be eradicated from newly introduced areas, thus preventing further expansion of their range. Because of long latent periods in humans (5∼15 years; Romig, 2003), surveillance of human population for the cases of alveolar echinococcosis would be insufficient. Active surveillance of wild hosts is needed for early detection of the parasite.

However, even in the regions where *E. multilocularis* is highly endemic, the prevalence among wildlife hosts is highly heterogenous with very low prevalence in most locations (Liccioli et al., 2014). For effective surveillance, wild hosts need be surveyed extensively at the scale of landscape. Due to transportation by humans, the potential area of new introduction is not limited to areas immediately adjacent to the current distribution but over any areas with potential hosts (Santa et al., 2021). Surveillance of wildlife over such vast areas is impractical.

Like most Taeniids, *E. multilocularis* has a complex life-cycle requiring two hosts of different species to complete life-cycle. In addition, the parasite has free-living stage outside of a host as embryonated eggs. The eggs can be found in environmental mediums such as water, soil, and on surfaces of vegetables, fruits, and mushrooms (Szostakowska et al., 2014; Lass et al., 2015; Lass et al., 2019; Barlaam et al., 2021; Saelens, Robertson & Gabriël, 2022). Most of the study on detection of Taeniids in environmental mediums were aimed at finding potential transmission route by seeking intact eggs, and were often processed through filtration, sedimentation and flotation for isolating the eggs and visual confirmation by microscope, before using PCR to confirm the species (Saelens, Robertson & Gabriël, 2022). These studies would not detect DNA of the parasite from sources other than intact eggs, such as those that might be found in bloods and carcasses of intermediate hosts, adult worms that ejected from definitive hosts after it died or failed to attach to the intestinal walls, or remains of eggs. To the best of our knowledge, no study has been conducted on *E. multilocularis* by searching its DNA in environmental medium as a mean for surveillance.

In this pilot study, we assessed whether *E. multilocularis* can be detected from standard eDNA samples. We used eDNA samples that were collected from rivers in known *E. multilocularis* endemic areas of Japan, with original purpose of surveillance study of fish species. If the parasite’s DNA can be detected using typical eDNA samples, surveillance of the parasite can be conducted alongside other biological surveys in future, greatly reducing the costs and efforts.

## Materials & Methods

### Study area & sample preparation

**Figure 1:**
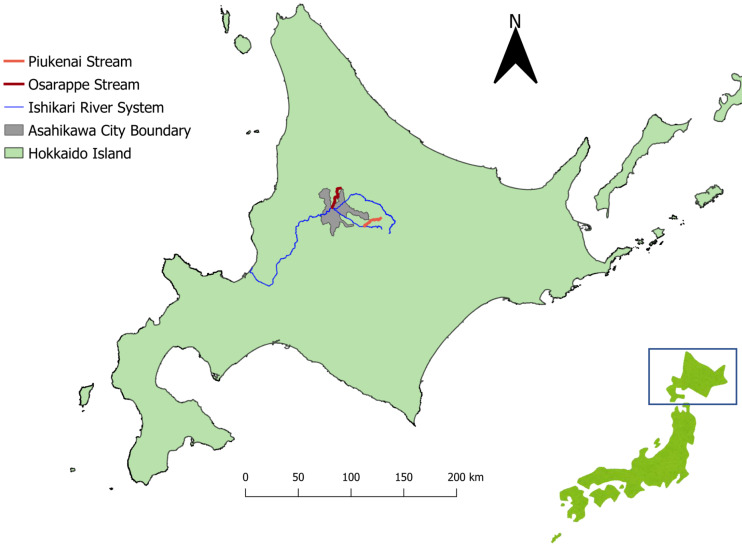
Map of the sampling sites. The streams where we took eDNA samples were located in central part of Hokkaido, Japan, where *Echinococcus multilocularis* is known to be endemic. Three sampling sites were assigned each to the three streams of Osarappe, Piukenai, and one of the tributaries of Ishikari river. Seven of the sampling sites were located at manmade structures where samples were taken on immediately upstream and downstream, thus totaling 16 samples at a time. The samples were taken eight times from October 2016 to November 2017. The map was produced with QGIS version 3.22.3 using geographical data produced by National Land Information Division, National Spatial Planning and Regional Policy Bureau, Ministry of Land, Infrastructure, Transport and Tourism of Japan (https://nlftp.mlit.go.jp/ksj/index.html).

The eDNA samples were extracted from river waters, with original purpose of surveying the distribution of salmonids (Imamura et al., 2019; Minamoto et al., 2019). The samples were collected from streams (Osarappe, Piukenai, and a tributary of Ishikari River) that flow into Ishikari River in Hokkaido, Japan (Fig. 1). The areas surrounding these streams are inhabited by foxes, a known definitive host of *E. multilocularis*. Three sampling sites were placed in each of the three streams, seven of which were at manmade structures where two samples were collected up- and downstream of the structures (Table 1). The sampling were conducted eight times in 2016–17, totaling 128 samples (details described in Minamoto et al., 2019).

**Table 1:** Summary of the sample sites. The sample water was taken from these sites eight times each, in October of 2016 and March, May, June, July, August, September and November of 2017. Where there was manmade structure, a sample was taken upstream and downstream of the structure each time.

	Stream	Distance from river mouth of each stream system (km)	Presence of manmade structure	Altitude (m)
Site 1	Tributary of Ishikari River	18.9	Yes	1020
Site 2			10.9	Yes	450
Site 3		10.4	Yes	440
Site 4	Piukenai Stream	2.4	Yes	520
Site 5			1.2	Yes	470
Site 6		0.4	No	440
Site 7	Osarappe Stream	20.3	Yes	150
Site 8			13.3	Yes	123
Site 9		0.28	No	105

Each sampling collected approximately 900 ml surface water, which were mixed with 1 ml of 10% Benzalkonium chloride solution to prevent DNA degradation (Yamanaka et al., 2017). The collected waters were filtered through glass fiber filters (pore size 0.7 µm; GF/F, GE Healthcare, Chicago, IL, USA) on the day of collection. Another 900 ml pure water was similarly filtered on the sampling day as a filtration blank. The filter papers were then stored at −25 °C until next process. The filters were then incubated with 400 µl of Buffer AL (Qiagen, Hilden, Germany) and 40 µl of Proteinase K (Qiagen) in Salivette tubes (Sarstedt, Nümbrecht, Germany) at 56 °C for 30 min, then centrifuged at 3,000 × g for 3 min. After centrifugation, 300 µl of Tris-EDTA (TE) buffer was added, and centrifuged again at 3,000 × g for 1 min. The DNA was extracted from the elution using the DNeasy Blood & Tissue Kit (Qiagen) following the manufacturer’s protocol. The final DNA samples were then stored at −25 °C. Positive control of *E. multilocularis* DNA was prepared from a sample of protoscolices isolated by Faculty of Veterinary Medicine, Disease Control group of Hokkaido University. Its DNA was extracted using DNeasy Blood & Tissue Kit (Qiagen) following the manufacturer’s protocol.

The concentration of DNA in the product was measured using Invitrogen Qubit3 (Thermo Fisher Scientific, Waltham, MA, USA).

### Primer-probe design

Primers and a probe were newly designed to detect eDNA, which are often in shorter fragments, and to accommodate *E. multilocularis* strain in Japan. The primer-probe targeted COB region of the mitochondrial DNA found on National Center for Biotechnology Information (NCBI) nucleotide database (accession numbers AB461399, AB374426, and AB477009; See Supplementary Material for the full targeted sequence). The primers-probes were; EmCOB; forward: 5′-AGTTATGGGTGAGGCATTTACTGGT-3′, reverse: 5′-CATAGAACCAACCAACGGCAAACT-3′, and probe: 5′-CTTATTGGGCTGCCACTG-3′. We determined the specificity of this primer-probe set in silica using the NCBI Primer Blast. Although the primers potentially amplify other species of the genus *Echinococcus*, other *Echinococcus* species are not known in Japan. More importantly, the probe was specific to *E. multilocularis*. Therefore we determined the primer-probe to be specific to the parasite.

The sensitivity of the primer-probe in detecting *E. multilocularis* (limit of detection: LOD) was assessed *in vitro* using the DNA extract from the protoscolices. The DNA of the was first diluted with TE buffer (Thermo Fisher Scientific) to standardized concentrations of 0.5, 0.05, 0.005, 0.0005, and 0.0005 pg/µl, and then assessed with real-time PCR.

The specificity of the primer-probe was assessed *in vitro* against a sample of another taeniid species (*Taenia taeniaeformis*), isolated by Azabu University Laboratory of Environmental Biology, that was potentially present in Japan. The DNA of the *T. taeniaeformis* were extracted using DNeasy Blood & Tissue Kit (Qiagen) following the manufacturer’s protocol, and the DNA concentration were standardized to 0.5, 0.05, and 0.005 pg/µl using Invitrogen Qubit3 (Thermo Fisher Scientific, Waltham, MA, USA). The real-time PCR of the *T. taeniaeformis* with the primer-probe did not result in any amplification at any of the concentrations.

### Real-time PCR

The real-time PCR was conducted using the Applied Biosystems StepOnePlus Real-Time PCR System (Thermo Fisher Scientific). Each reaction well was prepared by mixing 2 µl of the eDNA sample with 10µl of TaqMan Environmental Master Mix 2.0 (Thermo Fisher Scientific), 0.1 µl of AmpEraseUracil N-Glycosylase (Thermo Fisher Scientific), 9 µl of distilled water, and 1 µl of primer-probe mix. The primer-probe mix consists of 18 µM forward and reverse primers and 2.5 µM probe. The solution was first placed in 50 °C for 2 min, followed by 95 °C for 10 min, followed by 55 cycles of 95 °C for 15 s and 60 °C for 60 s.

Real-time PCR was repeated three times for each sample, along with filtration blank, positive control and negative control. The PCR products with amplification response were then assessed using Sanger sequencing. Sections of the resulting sequences with low quality base scores (<Q20) were trimmed off using Chromatogram Explorer (Heracle BioSoft SRL Mioveni, Romania). Then matching sequences were searched on NCBI nucleotide blast.

## Results

The EmCOB primer-probe successfully amplified DNA extracted from *E. multilocularis* protoscolices sample using real-time PCR. The PCRs using the parasite DNA of 1, 0.1, and 0.01 pg/reaction all showed amplification in all three repeats, with mean CT of 32.42, 35.80, and 38.72 respectively. PCR with DNA of 0.001 pg/reaction resulted in no amplification in any of the three repeats. Therefore, we determined the LOD of this primer-probe to be 0.01 pg/reaction.

One of the samples taken from Piukenai stream, collected at the mid-point of three sampling sites of that stream in March of 2017, had one replicate that was positive for *E. multilocularis* (CT 39.93). The Sanger sequencing results of the positive PCR product confirmed that the amplified DNA sequence was that of *E. multilocularis* (see Supplementary Material). Six other samples had at least one repeat with amplification above 40 CT, but the amplification was much weaker than that of positive control. The Sanger sequencing results of these PCR products did not match the sequence of *E. multilocularis* and were therefore considered all negative. The positivity rate by samples was 0.78% (1 / 128).

## Discussion

In this study we successfully detected DNA of *E. multilocularis* in an eDNA samples originally collected for a study of another species, despite at a very low rate. To the best of our knowledge, this is the first study that found the DNA of *E. multilocularis* in environmental mediums using standard eDNA methods. Given the increasing number of eDNA studies on various taxa and given the concerns for expanding distribution of the parasite, the result of this study is potentially significant. However, this study also indicates that detectability of the parasite using eDNA is rather low. We did not detect the parasite’s DNA in the other repeats of the same samples, in the samples taken from the same stream, or in the samples taken from the same location at different times. It is possible that the prevalence of the parasite in the sampled area is low, but for the purpose of surveillance or early detection we would hope to find the parasite at low prevalence.

Even the sample with which we found the DNA of *E. multilocularis* seemed to have very low concentration. The CT value of 39.93 of the positive sample indicate that the DNA of the parasite in the sample was at a concentration below 0.01 pg/reaction which had mean CT value of 38.72. In other words, the sample had DNA concentration that was barely detectable with this primer-probe set, likely the reason why two of the three replicates from this sample failed to amplify with the real-time PCR.

The use of repurposed eDNA for early detection of *E. multilocularis* seems unpractical at the low rate of detection shown in this study. However, the prevalence of the parasite among wildlife hosts can naturally be quite low. For example, in the City of Calgary, Canada, where the overall prevalence of the parasite among definitive hosts was high (21.42%), the prevalence assessed by smaller areas within the city often showed much lower prevalences (5∼6%; Liccioli et al., 2014). The prevalence among intermediate hosts was even lower (0.6∼1.4%), and often not found in many parts of the city (Liccioli et al., 2014). Therefore, even with a targeted survey on wildlife hosts, large quantities of samples are required to reliably detect the parasite. Considering the costs and efforts of collecting the samples, the eDNAs still hold some advantages over wildlife surveys, especially if the eDNA samples are already available from other studies. If the methodologies can be modified to improve the detection rate, eDNA can be a very practical tool for public health.

One factor that likely reduced the detection rate of *E. multilocularis* is the DNA extraction method. The primary presence of the parasite in the environment is likely the eggs. Extraction of DNA from the taeniid eggs are known to be impeded by robust egg case, and processes that physically break the egg case before extraction is often needed for an accurate PCR result (Klein, Liccioli & Massolo, 2014; Bass et al., 2015). As such processes may cause degradation of other DNAs in the samples (Shao, Khin & Kopp, 2012), this can be a significant drawback for sharing or repurposing samples with other studies. In addition, Lass et al. (2019) found only 1.9% positive in environmental water using extraction methods designed for the parasite’s eggs, suggesting that changing the extraction method may not greatly increase the detection rate in water.

The DNA of *E. multilocularis* is found in water likely because eggs or tissues of the parasite were washed down from their original location and their presence is expected to be ephemeral, especially in lotic water. It is also noteworthy that Lass et al. (2019) found the parasite only in lake waters and not in river waters. Szostakowska et al. (2014) found a higher positivity rate (11.3%) from soil samples, suggesting soil as better medium for detecting the parasite. However, eDNA from soil would have small spatial coverage per sample as it would require the parasite’s DNA to be directly deposited onto that soil, as opposed to water which can gather parasite’s DNA from potentially entire watershed through water flow.

Future study should assess the detectability of *E. multilocularis* using eDNA method by sampling wild hosts alongside sampling environmental mediums. Knowing the prevalences and infection intensities among wild hosts in the immediate vicinity of the eDNA samples can provide us with better grasp of the detectability of the parasite using eDNA methodology.

One advantage of using eDNA for *E. multilocularis* and other parasites is that the eDNA samples potentially contain information of entire biota in the area, including the terrestrial host species and its community composition even from water samples (Ushio et al., 2017). Such information can in turn be used to predict the areas of high risk (Mori et al., 2019). Environmental DNA may also provide interesting insight to the parasites communities as a whole and their interrelations with *E. multilocularis*.

## Conclusions

In this study we sought to determine if *E. multilocularis* can be detected in repurposed eDNA samples originally collected for another purpose. Though we were successful in finding DNA sequence of *E. multilocularis* in the repurposed eDNA samples, the chance of detecting the parasite appears to be low. Further study is desired to assess and improve the methods before we can claim the method to be practical.

##  Supplemental Information

10.7717/peerj.15431/supp-1 Supplemental Information 1 Realtime PCR results Raw output data from StepOnePlus. Each sheet shows results from all the PCR on samples tested at one batch. First two sheets are the results of testing the primer-probe on the *Echinococcus multilocularis* positive control (labelled as Em followed by its concentration in pg/µl) and DNA extracted from *Taenia taeniaeformis* (labelled as Tt followed by its concentration in pg/µl). Negative control is labelled as NC. Third sheet is the results of testing the limit of detection of the *E. multilocularis* on this primer-probe system.
Remaining sheets are the results of PCR on river water samples. The samples are labelled with Osh and three digit identifiers. The first digit indicate the sampling session (1–8), the other two digits indicate the sampling sites (1–16 or NC for filtration blank).

10.7717/peerj.15431/supp-2 Supplemental Information 2 Target sequence of the primers Sequence of COB region of the mitochondrial DNA of *Echinococcus multilocularis* that were targeted by the primers in this study

10.7717/peerj.15431/supp-3 Supplemental Information 3 Results of Sanger sequencing of the PCR product that showed amplification of *Echinococcus multilocularis* DNA Sections with low quality base scores (<Q20) were trimmed off using Chromatogram Explorer (Heracle BioSoft SRL 2020).

## Additional Information and Declarations

### Field Study Permissions

The following information was supplied relating to field study approvals (*i.e.*, approving body and any reference numbers): Under Japanese law, river water can be collected without permission. No permission/approval is required for sample collection in this study.

### Data Availability

The following information was supplied regarding data availability: The raw data is available in the Supplemental Files.
